# Internal Fixation Versus Nonoperative Treatment for Displaced 3-Part or 4-Part Proximal Humeral Fractures in Elderly Patients: A Meta-Analysis of Randomized Controlled Trials

**DOI:** 10.1371/journal.pone.0075464

**Published:** 2013-09-16

**Authors:** Yongchuan Li, Liangyu Zhao, Lei Zhu, Jing Li, Aimin Chen

**Affiliations:** Department of Orthopaedic Trauma Surgery, Orthopaedic Institute of PLA, Shanghai Changzheng Hospital, Second Military Medical University, Shanghai, China; Faculté de médecine de Nantes, France

## Abstract

**Background:**

A few studies focused on open reduction and internal fixation (ORIF) or nonoperative treatment of displaced 3-part or 4-part proximal humeral fractures in elderly patients have been published, all of whom had a low number of patients. In this meta-analysis of randomized controlled trials (RCTs), we aimed to assess the effect of ORIF or nonoperative treatment of displaced 3-part or 4-part proximal humeral fractures in elderly patients on the clinical outcomes and re-evaluate of the potential benefits of conservative treatment.

**Methods:**

We searched PubMed and the Cochrane Central Register of Controlled Trials databases for randomized controlled trials comparing ORIF and nonoperative treatment of displaced 3-part or 4-part proximal humeral fractures in elderly patients. Our outcome measures were the Constant scores.

Results: Three randomized controlled trials with a total of 130 patients were identified and analyzed. The overall results based on fixed-effect model did not support the treatment of open reduction and internal fixation to improve the functional outcome when compared with nonoperative treatment for treating elderly patients with displaced 3-part or 4-part proximal humeral fractures (WMD −0.51, 95% CI: −7.25 to 6.22, P = 0.88, I^2^ = 0%).

**Conclusions:**

Although our meta-analysis did not support the treatment of open reduction and internal fixation to improve the functional outcome when compared with nonoperative treatment for treating elderly patients with displaced 3-part or 4-part proximal humeral fractures, this result must be considered in the context of variable patient demographics. Only a limited recommendation can be made based on current data. Considering the limitations of included studies, a large, well designed trial that incorporates the evaluation of clinically relevant outcomes in participants with different underlying risks of shoulder function is required to more adequately assess the role for ORIF or nonoperative treatment.

## Introduction

Proximal humeral fracture is one of the most frequent osteoporotic fractures in the elderly people, accounting for 6% of all fractures seen in accident and emergency departments[1]–[3]. Most proximal humeral fractures are undisplaced or minimally displaced[4] and can be treated successfully nonoperatively[5]. The most frequently used classification for proximal humeral fractures is the Neer classification[6], [7] which is based on the 4 anatomical segments of the proximal humerus (the humeral head, shaft, greater and lesser tubercles) and whether these segments are fractured and displaced.

According to Neer classification, 3-part and 4-part proximal humeral fractures are comminuted displaced fractures which represent 13% to 16% of all proximal humeral fractures[1]. Operative treatment of these fractures in younger patients is not controversial. The main controversy pertains to elderly patients with varying degrees of osteoporosis and 3-part or 4-part proximal humeral fractures after low-energy trauma. Whether these fractures need surgery or not, remains controversial.

A few studies[8]–[10] focused on open reduction and internal fixation (ORIF) or nonoperative treatment of displaced 3-part or 4-part proximal humeral fractures in elderly patients have been published, all of whom had a low number of patients. In this meta-analysis of randomized controlled trials (RCTs), we aimed to assess the effect of ORIF or nonoperative treatment of displaced 3-part or 4-part proximal humeral fractures in elderly patients on the clinical outcomes and re-evaluate of the potential benefits of conservative treatment.

## Materials and Methods

### Search strategy

The literature search was performed on PubMed (1966- October 2012), and the Cochrane Central Register of Controlled Trials (1996 to October 2012). We used the following search terms in different combinations as MeSH (Medical Subject Heading) terms and as text words: Proximal humeral fracture, internal fixation, treatment outcome, surgery and comparative study. We did not restrict by language or type of article. To identify other relevant studies, we manually scanned reference lists from identified trials and review articles, and we also searched conference proceedings. We requested original data by directly contacting authors.

### Study selection

We included studies when the following criteria were met: (1) randomized, controlled trials assessing treatment for displaced 3-part or 4-part proximal humeral fractures in elderly patients; (2) the intervention was open reduction and internal fixation versus nonoperative treatment; (3) studies reported the the outcome measure of the Constant score in both arms. The primary outcome measure was the Constant score, which is the most often used functional score as an outcome measure in studies of proximal humeral fractures[11]. Secondary outcome measures were nonunion, avascular necrosis (AVN) of humeral head and osteoarthritis.

### Data extraction and quality assessment

Data were collected independently by 2 reviewers. Extracted data included patient characteristics (mean age, female rate); inclusion criteria; protocol for the treatment of fractures; Constant score and complications. Quality assessment was judged on concealment of treatment allocation; similarity of both groups at baseline regarding prognostic factors; eligibility criteria; blinding of outcome assessors, care providers, and patients; completeness of follow-up; and intention-to-treat analysis[12]. We quantified study quality by using the Jadad score[13]. A third reviewer adjudicated any disagreement about extracted data. Then data were checked and entered into the Review Manager (Version 5.0. Copenhagen: The Nordic Cochrane Centre, The Cochrane Collaboration, 2008) database for further analysis.

### Statistical analysis

Continuous variable (the Constant Score) was analysed using the weighted mean differences (WMD) with its 95% CI, whereas dichotomous data (nonunion, avascular necrosis of humeral head, and osteoarthritis) were analyzed using the risk ratio (RR) measure and its 95% confidence interval (CI). Moreover, heterogeneity across trials was evaluated with I^2^ statistic, which defined as I^2^>50%. If heterogeneity existed, a random-effect model was used to assess the overall estimate. Otherwise, a fixed-effect model was chosen. Sensitivity analyses (exclusion of one study at a time) were conducted to assess heterogeneity and robustness of pooled results. We assessed for potential publication bias by using the Begg adjusted-rank correlation test[14] and Egger regression asymmetry test[15]. All tests were two-tailed and a P value less than 0.05 was regarded as significant in this meta-analysis.

## Results

### Selected studies and characteristics

**Figure 1:**
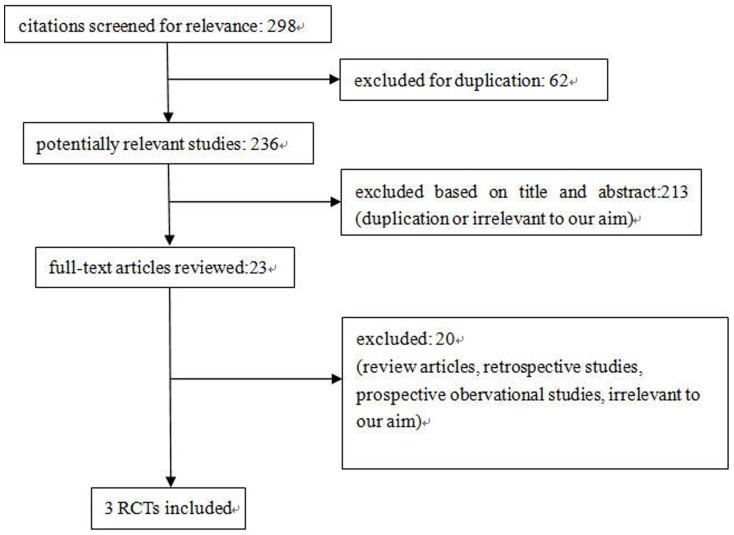
Study selection diagram.

We identified 298 potentially relevant citations from the initial literature search. After independently reviewing the title and abstract of all potential articles, 23 articles were considered of interest and reviewed in full-text. Of these, 19 were excluded from the meta-analysis (review articles, retrospective studies, prospective obervational studies, irrelevant to our aim). Although the study carried out by Fjalestad Tore et al[9] did not provide data on the Standard Deviation (SD) of Constant score, we requested it by directly contacting the author. Therefore, three randomized controlled studies with a total of 130 elderly patients with displaced 3-part or 4-part proximal humeral fractures were identified and analyzed[8]–[10]. Our search strategy is outlined in **Figure 1**.

**Table 1** and **table 2** summarizes the characteristics of the included studies. One of them[8] was reported in 1997 and others[9], [10] were reported in the past two years. 64 subjects were assigned to open reduction and internal fixation group and 66 subjects were assigned to nonoperative treatment group. All studies involved patients with displaced 3-part or 4-part proximal humeral fractures who were aged 55 years or older. Most subjects were females. Open reduction and internal fixation in one study[8] was treated by tension-band and in other two studies[9], [10] were treated by locking plate. Nonoperative treatment in three studies were almost same. The proportion of patients lost to follow-up was very high (27.5%) in the study carried out by Zyto Karol et al[8] and less than 10% in other two studies[9], [10]. Constant score was calculated in each study as the shoulder function outcome measure. Although the study carried out by Fjalestad Tore et al[9] did not provide data on the SD of Constant score, we requested it by directly contacting the author. The quality characteristics of the studies were shown in **table 3**.

**Table 1:** Characteristics of included studies.

Author, year	Patients,n		Female proportion in each group,%		Mean age,y		Three-part		Inclusion criteria
	Surgical	Conservative	Surgical	Conservative	Surgical	Conservative	Surgical	Conservative	
Zyto K, 1997	14	15	90	85	73	75	19	18	A displaced three- or four-part fracture of the humerus not caused by high-energy trauma and not pathological; at least 30% contact between the humeral head and the humeral shaft; no other fractures elsewhere in the upper limbs; no concomitant disease likely to influence the end result; and ability of the patient to co-operate
Fjalestad T, 2011	23	25	80	96	72.2	73.1	\	\	Patients aged 60+ years with a displaced, unstable three- or four-part proximal humerus fracture of OTA group 11-B2 or 11-C2 (displaced fracture of extra-articular or articular, bifocal type) were included in this study
Olerud P, 2011	27	26	80	83	72.9	74.9	30	30	A displaced 3-part fracture of the proximal humerus in elderly patients. The patient inclusion criteria were age 55 or older, a fracture sustained after a low-energy trauma (ie, simple fall), no previous shoulder problems, independent living conditions (ie, not institutionalized), and no severe cognitive dysfunction (ie, 3 correct answers on a 10-item Short Portable Mental Status Questionnaire [SPMSQ]).

**Table 2:** Characteristics of included studies-continued.

Surgical treatment	Conservative treatment	Constant score		Complications		Follow-up time
		Surgical	Conservative	Surgical	Conservative	
Tension-band surgery	Conservative treatment	60±19	65±15	AVN 1, nonunion 1, osteoarthritis 4	osteoarthritis 2	50 months
Open reduction and internal fixation using an angular stable plate and cerclages	Standardized nonoperative treatment	52.3±20.9	52.2±18.3	AVN 8, nonunion 0	AVN 13, nonunion 2	1 year
ORIF with a locking plate	Nonoperative treatment	61±19.2	58.4±23.1	AVN 3, nonunion 1, osteoarthritis 0	nonunion 1, AVN 2, osteoarthritis 1	2 years

**Table 3:** Quality of included RCTs.

Author, year	Jadad Score	Allocation Concealment	Similarity of Baseline Characteristics	Eligibility Criteria	Blinding			Completeness of Follow-up	Intention-to-Treat Analysis
					Outcome Assessor	Care Provider	Patient		
Zyto K, 1997	1+0+1	YES	YES	YES	YES	NS	NS	YES	NS
Fjalestad T, 2011	2+0+1	YES	YES	YES	NO	NS	NS	YES	YES
Olerud P, 2011	1+0+1	NS	YES	YES	YES	NS	NS	YES	YES

NS = not specified or available.

### Effects of ORIF vs nonoperative treatment

**Figure 2:**
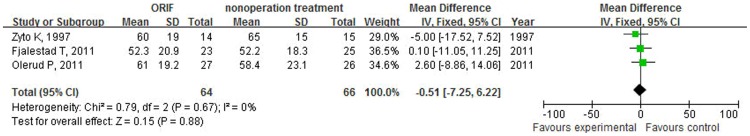
Forest plot of mean difference and 95% confidence intervals (CI) for constant scores among patients assigned to ORIF versus nonoperative treatment.

**Figure 3:**
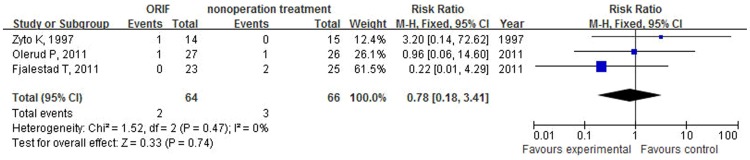
Forest plot of risk ratios and 95% confidence intervals (CI) for the incidence of nonunion among patients assigned to ORIF versus nonoperative treatment.

**Figure 4:**
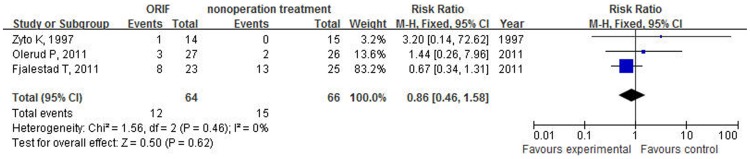
Forest plot of risk ratios and 95% confidence intervals (CI) for the incidence of AVN among patients assigned to ORIF versus nonoperative treatment.

**Figure 5:**
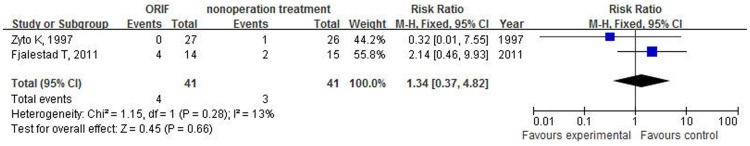
Forest plot of risk ratios and 95% confidence intervals (CI) for the incidence of osteoarthritis among patients assigned to ORIF versus nonoperative treatment.

Three studies[8]–[10] that included 130 cases provided data on Constant score. The overall results based on fixed-effect model did not support the treatment of open reduction and internal fixation to improve the functional outcome when compared with nonoperative treatment for treating elderly patients with displaced 3-part or 4-part proximal humeral fractures (WMD −0.51, 95% CI: −7.25 to 6.22, P = 0.88, I^2^ = 0%, **figure 2**). Incidence of nonunion was low in both arms and there was not significant different after the use of ORIF compared with nonoperative treatment (RR = 0.78; 95% CI: 0.18–3.41, P = 0.74, I^2^ = 0%, **figure 3**). Nonoperative treatment was not associated with a significant reduction in risk of avascular necrosis of humeral head (RR = 0.86; 95% CI: 0.46–1.58, P = 0.62, I^2^ = 0%, **figure 4**). Two studies reported data on osteoarthritis, including 82 patients. Fractures receiving ORIF did not show more osteoarthritis than those receiving nonoperative treatment (RR = 1.34; 95% CI: 0.37–4.82, P = 0.66, I^2^ = 0%, **figure 5**).

Assessment of publication bias using Egger's and Begg's tests showed that no potential publication bias existed among the included trials (Egger's test: p>0.05; Begg's test: p>0.05).

## Discussion

To our knowledge, this is the first meta-analysis of randomized controlled trials comparing open reduction and internal fixation and nonoperative in the treatment of displaced 3-part or 4-part proximal humeral fractures in elderly patients. This meta-analysis was based on 3 randomized controlled trials that included 64 fractures treated with ORIF and 66 fractures treated with nonoperative. The outcomes investigated were shoulder function (Constant Score), nonunion, avascular necrosis and osteoarthritis. Our results failed to show beneficial effects of ORIF on the shoulder function measured by Constant score, on the risk of nonunion, avascular necrosis of humeral head or osteoarthritis, compared with nonoperative treatment. Because of poor number included among these studies, a large definitive RCT is needed.

Almost 40 years ago, early mobilisation was attentioned by orthopedic doctors. With the development of better fixation devices, open reduction and internal fixation in treating elderly patients with displaced 3-part or 4-part proximal humeral fractures is increasingly popular. There are several predictors of success with surgical treatment of a proximal humeral fracture such as careful soft tissue management, anatomic reduction, and proper plate placement. However, we surgeons can not controll everything. For instance, primary devascularization of the head fragments resulting from the injury itself, particularly in severely displaced Type B fractures and in most displaced C fractures, is an important problem that cannot be controlled[16]. In this meta-analysis, open reduction and internal fixation in one study[8] was treated by tension-band and in other two studies[9], [10] were treated by locking plate. We did not find better shoulder function, lower rate nonunion, higher rate avascular necrosis of humeral head and higher rate osteoarthritis, compared with nonoperative treatment. This may be explained partly by above predictors.

In addition to open reduction and internal fixation, shoulder arthroplasty might be an alternative surgical method for treating elderly patients with displaced 3-part or 4-part proximal humeral fractures. Three different arthroplasty are available: hemiarthroplasty (HAS), total shoulder arthroplasty (TSA) and reverse shoulder arthroplasty (RSA). HAS and TSA are indicated for patients with intact rotator cuffs. RSA is indicated for elderly patients with massive irreparable rotator cuff tears and is usually considered as a ‘‘last chance’’ possible surgery for a functional shoulder[17], [18]. For these elderly displaced 3-part or 4-part proximal humeral fracture patients with seriously rotator cuff tears, RSA should be a suitable method instead of ORIF which might have been used in these patients with poor shoulder function. Although RSA shoulders have showed better functional improvement in a limited number of studies, they have also demonstrated higher complication rates[19]–[21]. Moreover, the RSA shoulder implant is also markedly more expensive than other methods'. Because of these, cost-effectiveness analysis can be used to evaluate the decision about which arthroplasty or ORIF is preferred and related study is needed to be carried out.

Recent studies have shown the significant complication rate associated with operative treatment of above injuries[22], [23]. As such, nonoperative treatment has been re-emphasized these days. Yuksel HY et al[24] carried out a study to evaluate the results of nonoperative treatment of three- and four-part fractures of the proximal humerus in patients who refused surgery or could not undergo surgery because of medical conditions and found satisfactor results either in younger or elderly patients. Iyengar JJ et al[25] did a systematic review of nonoperative management of proximal humerus fractures which demonstrated high rates of radiographic healing, good functional outcomes, and a modest complication rate. Patients with nonoperative treatment in the studies included in our meta-analysis all received standardized treatment. We did not found a significant difference compared with ORIF treatment, which is in line with previous studies.

The updated Cochrane meta-analysis published in 2012 was unable to provide guidelines for treating proximal humerus fractures due to a lack of solid evidence. Indeed, meta-analysis of Handoll et al published in 2012 is a detailed, wide and good paper. In this paper, the authors gave us lots of data about interventions for treating proximal humeral fractures in adults, such as comparison of early mobilisation versus immobilisation for 3 weeks, comparison of surgery versus conservative treatment, comparison of locking plate versus locking intramedullary nail and comparison of hemi-arthroplasty versus tension band wiring and so on. However, our meta-analysis is specifically focused on the problem of ORIF vs non operative treatment for treating elderly patients with displaced 3-part or 4-part proximal humeral fractures. We analyzed the data and made the recommendation and this may be a reference for our clinical doctors that we can consider conservative treatment instead of ORIF.

There are several potential limitations in this meta-analysis. Firstly, one prominent drawback pertinent to this study is that only three RCTs with 130 subjects were included in this meta-analysis; the results of pooled analysis might therefore be accompanied with bias. Secondly, although all included studies reported the Constant score, few trials designed to investigate the effect of ORIF on some clinical outcomes such as malunion, osteoarthritis, axillary nerve injury and infections. Finally, we did not have access to patient-level data to determine whether the risk factors (eg, gender and age) could influence the effect of open reduction and internal fixation on the shoulder function.

In conclusion, although our meta-analysis did not support the treatment of open reduction and internal fixation to improve the functional outcome when compared with nonoperative treatment for treating elderly patients with displaced 3-part or 4-part proximal humeral fractures, this result must be considered in the context of variable patient demographics. Only a limited recommendation can be made based on current data. Considering the limitations of included studies, a large, well designed trial that incorporates the evaluation of clinically relevant outcomes in participants with different underlying risks of shoulder function is required to more adequately assess the role for ORIF or nonoperative treatment.

## Supporting Information

Checklist S1 PRISMA Checklist
(DOC)

Diagram S1 PRISMA Flow Diagram
(DOC)
