# Supplementary material for: Internal Fixation Versus Nonoperative Treatment for Displaced 3-Part or 4-Part Proximal Humeral Fractures in Elderly Patients: A Meta-Analysis of Randomized Controlled Trials
Source: PLoS One. 2013 Sep 16;8(9):e75464. doi: 10.1371/journal.pone.0075464 (PMC3774627; doi:10.1371/journal.pone.0075464)
Supplement: Diagram S1 — PRISMA Flow Diagram (DOC) [file pone.0075464.s002.doc]

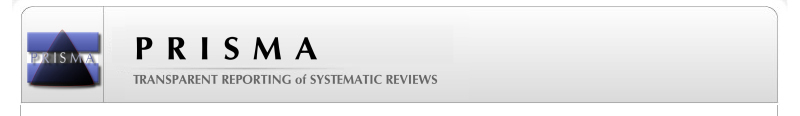
**PRISMA 2009 Flow Diagram**

**Identification**

citations screened for relevance: 298

excluded for duplication: 62

potentially relevant studies: 236

excluded based on title and abstract: 213 (duplication or irrelevant to our aim)

full-text articles reviewed:23

excluded: 20

(review articles, retrospective studies, prospective obervational studies, irrelevant to our aim)

3 RCTs included

**Screening**

**Eligibility**

**Included**

Studies included in quantitative synthesis (meta-analysis)
(n = )
